# The Vasomotor Nerves of the Pia Mater and Brain

**Published:** 1868-03

**Authors:** H. Nothnagel

**Affiliations:** Assist.-Physician to the Medical Policlinic at Könisberg, Prussia


### THE VASOMOTOR NERVES OF THE PIA MATER AND BRAIN.

By Dr. H. NOTHNAGEL,
Assist.-Physician to the Medical Policlinic at Könisberg, Prussia.

[Virchow's Archiv. XL., 203. 1867.]

New experimental investigations (on rabbits) have led the author to these results:

(1.) The vasomotor nerve-fibres for the vessels of the pia mater partly belong to the ganglionic chain of the cervical part of the sympathetic.

(2.) Another, perhaps more considerable part, of them enter the superior cervical ganglion.

(3.) Some take a course above this ganglion even, very probably in cerebral nerves.

The same results can safely be predicated of the vessels of the cerebral substance itself, though direct observation is here impossible.

These vivisectional experiments have further shown, that violent irritations (electrical or mechanical) of sensory nerves cause contraction in the arteries of the pia mater. A rabbit having been trephined and the dura mater removed, if the metallic electrodes of a strong inductive apparatus be placed upon the thigh at points corresponding to the course of the crural nerve, a distinct contraction of the arteries of the pia mater will be observed, which will continue for a few minutes after the removal of the electrodes, and gradually pass into dilatation. Undoubtedly this contraction of the arteries is of reflex nature. The excitation is transferred, in the medulla oblongata, to the vasamotor nerves of the head. Partial interruption of the courses of the latter, as far as that is possible, diminishes the effect of the irritation to a minimum. Severe mechanical irritation of the leg had the same effect.

This reflex contraction of the cerebral vessels is of importance in the analysis of the mechanism of the epileptic attack. Lately the opinion has gained ground, that a spasm of the cerebral arteries is the first link in the chain of phenomena constituting an epileptic or eclamptic paroxysm; this spasm causes anæmia of the brain, upon which depend the convulsions and coma (Kussmaul and Tenner, Brown-Séquard, Reynolds). Now it is certain, that there are cases of epilepsy that can safely be designated as "reflex epilepsy"—cases in which the disease arose from an injury or the like. The reflex origin of the convulsions is still more extended as concerns "acute epilepsy": eclampsia; (eclamptic attack of children in teething, from presence of worms, etc.) The author's experiments may perhaps throw some light upon this form of the disease. For if the above idea of the causation of epileptic attacks be correct, we must assume that in reflex epilepsy a contraction of the cerebral vessels takes place upon a peripheric sensory irritation. The experiments have actually demonstrated the occurrence of this reflex contraction of the arteries of the brain.—*St. Louis Med. & Surg. Jour.*

---

Blood-Corpuscles in Chlorosis.—M. Duncan, of St. Petersburg, has just pointed out the remarkable fact that the blood-discs of chlorotic persons yield up their coloring matter more easily than do those of healthy subjects.

